# The roles of N6-methyladenosine and its target regulatory noncoding RNAs in tumors: classification, mechanisms, and potential therapeutic implications

**DOI:** 10.1038/s12276-023-00944-y

**Published:** 2023-03-01

**Authors:** Ziying Liu, Lei Gao, Long Cheng, Gaoyuan Lv, Bei Sun, Gang Wang, Qiushi Tang

**Affiliations:** 1 Department of Pancreatic and Biliary Surgery, The First Affiliated Hospital of Harbin Medical University, Harbin, Heilongjiang China; 2 Key Laboratory of Hepatosplenic Surgery, Ministry of Education, The First Affiliated Hospital of Harbin Medical University, Harbin, Heilongjiang China; 3 Chinese Journal of Practical Surgery, Chinese Medical University, Shenyang, Liaoning China

**Keywords:** Tumour biomarkers, Targeted therapies

## Abstract

N6-methyladenosine (m6A) is one of the epigenetic modifications of RNA. The addition of this chemical mark to RNA molecules regulates gene expression by affecting the fate of the RNA molecules. This posttranscriptional RNA modification is reversible and regulated by methyltransferase “writers” and demethylase “erasers”. The fate of m6A-modified RNAs depends on the function of different “readers” that recognize and bind to them. Research on m6A methylation modification has recently increased due to its important role in regulating cancer progression. Noncoding RNAs (ncRNAs) are a class of RNA molecules that are transcribed from the genome but whose roles have been overlooked due to their lack of well-defined potential for translation into proteins or peptides. However, this misconception has now been completely overturned. ncRNAs regulate various diseases, especially tumors, and it has been confirmed that they play either tumor-promoting or tumor-suppressing roles in almost all types of tumors. In this review, we discuss the m6A modification of different types of ncRNA and summarize the mechanisms involved. Finally, we discuss the progress of research on clinical treatment and discuss the important significance of the m6A modification of ncRNAs in the clinical treatment of tumors.

## Non-coding RNAs: Modification by adding methyl groups

The reversible addition of a chemical methyl group (CH_3_) to the base adenine in RNA molecules, creating N6-methyladenosine, regulates the activity of many non-coding RNAs that, unlike messenger RNAs, do not code for the formation of proteins. These RNAs can regulate gene activity. Researchers in China led by Gang Wang at Harbin Medical University, and Qiushi Tang at the Chinese Medical University, Liaoning, review the molecular mechanisms and significance of ‘m6A modification’ of non-coding RNAs. They focus on the progress of research into the role of m6A modification in cancer and its potential relevance for diagnosis and treatment. Approaches to treating cancer by interfering with m6A modification have been explored in mice. Future research will aim to translate the promise of these early animal studies into tests in human cells and clinical trials in patients.

## Introduction

**Fig. 1:**
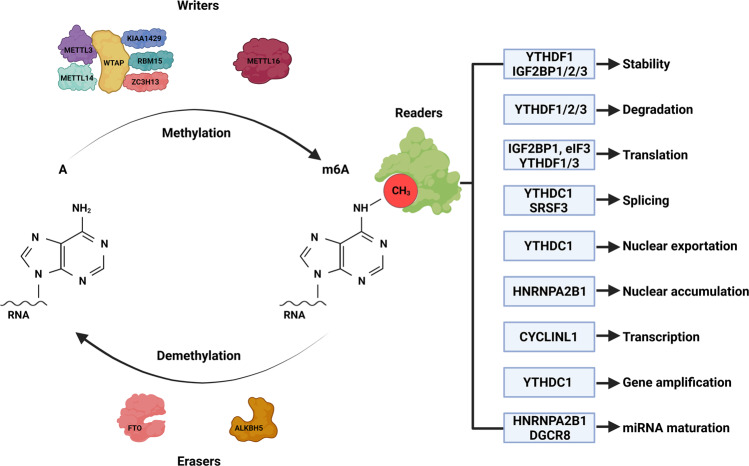
The structure and reversibility of m6A modifications and the function of m6A through recognition by the reader protein. m6A modification refers to the methylation of the sixth N atom of adenine in RNA molecules, which can be catalyzed by the MTC writing complex consisting of the core components of METTL3-METTL14-WTAP and other regulatory cofactors or by METTL16 alone. The m6A site on RNA can be recognized by the reader protein, causing changes in RNA function, and can also be reversibly removed by the erasure protein FTO or ALKBH5.

N6-methyladenosine refers to the methylation of the sixth N atom of adenine in RNA molecules (Fig. 1). This modification occurs near stop codons, in 5’- and 3’- untranslated regions, in long internal exons, and in the shared sequence RRACH (R = G/A and H = A/C/U)^1^. Among the numerous methods of RNA modification discovered to date, m6A methylation is the most common and abundant epigenetic modification of RNA. m6A was first discovered in mRNA in the 1970s^2^ and has since been found to be widespread in a variety of organisms, such as viruses^3^, yeast^4^, and plants^5^. The m6A modification of RNA is performed by “writers”, including methyltransferase-like 3 (METTL3), methyltransferase-like 14 (METTL14), Wilms tumor 1-associated protein (WTAP), KIAA1429, zinc finger CCCH domain-containing protein 13 (ZC3H13), and RNA-binding motif protein 15 (RBM15). METTL3 is the core of the complex and is responsible for catalyzing the methylation of the 6th atoms of adenine (A) in RNA^6^; METTL14 acts as an RNA binding scaffold to activate and enhance the catalytic activity of METTL3^7^; WTAP has no catalytic activity but promotes m6A methylation by recruiting METTL3 and METTL14^8^; RBM15 binds to METTL3 and WTAP and recruits them to specific RNA sites to promote m6A modification^9^; ZC3H13 binds to WTAP and allows its retention in nuclear speckles (NSs) to promote m6A modification^10^; and KIAA1429 guides regioselective methylation by recruiting the m6A methyltransferase complex (MTC) to 3’ UTRs and stop codons^11^. In addition, methyltransferase-like 15 (METTL15) and methyltransferase-like 16 (METTL16) do not belong to the MTC and independently catalyze m6A methylation. Methyltransferase-like 16 (METTL16), a methylase that was discovered later, promotes m6A modification of U6-snRNA and participates in RNA presplicing^12^. The erasing of m6A is catalyzed by Human AlkB homolog H5 (ALKBH5) and fat mass and obesity (FTO) of the α-ketoglutarate-dependent dioxygenase family, which are known as “erasers”; thus, this m6A modification is reversible^13,14^ (Fig. 1). FTO catalyzes demethylation via the oxidation of m6A to N6-hydroxymethyladenosine and N6-formyladenosine, followed by their hydrolysis to adenine; in contrast, ALKBH5 directly removes the m6A modification^15^. m6A “readers” can be divided into the following categories. Class I readers include those with the YTH domain, including YTHDC1/2 and YTHDF1/2/3. They recognize and bind to m6A-containing transcripts through the YTH domain^16^. Class II readers are heterogeneous ribonucleic acid proteins (hnRNPs), including hnRNP C and hnRNP A2B1, and they regulate the alternative splicing or processing of transcripts^17^. The IGF2BP family of proteins, IGF2BP1/2/3, are class III readers, and they share 6 RNA-binding domains, including 2 RNA recognition motifs and 4 KH domains^18^. Other readers include eukaryotic initiation factor 3 (EIF3)^19^, which promotes the translation of ncRNAs, and (human antigen R) HuR^20^, which affects transcript stability (Fig. 1). As mentioned earlier, m6A modification is an important way to regulate gene expression through the modification of multiple steps in mRNA processing. There is evidence that m6A regulates splicing of pre-mRNA^21^. The mRNA containing m6A was recognized by YTHDC1 and exported to the cytoplasm after splicing^22^. In the cytoplasm, m6A modification of mRNA affects the stability of mRNA^23^. In addition, m6A regulates mRNA translation, including translation initiation and translation elongation^24,25^. Here, we will review the ncRNAs in tumors that are regulated by these three classes of effectors and thus influence tumor progression. With the rapid development of sequencing technology, we found that m6A modification regulates ncRNA fate in many ways. m6A-modified RNAs regulate many pathological and physiological processes by controlling gene expression. Disorders of m6A modification are closely related to the occurrence and development of tumors^26^. In addition, some other RNA modification methods, such as N1-methyladenosine (m1A), 5-methylcytidine (m5C), N (7)-methylguanosine (m7G), and N4-acetylcytidine (ac4C), will also be briefly described at the end of this review.

ncRNAs are RNAs that are transcribed from the genome and were once thought to be byproducts of meaningless transcription because they are not translated into proteins. However, as research progressed, it was found that these “junk RNAs” are widely involved in important biological functions. ncRNAs comprise many species, many of which have been identified as markers of posttranscriptional regulation. Here, we mainly describe the three types of ncRNA that are most commonly modified by m6A, namely, microRNAs (miRNAs), long noncoding RNAs (LncRNAs) and circular RNAs (circRNAs). miRNAs are a class of ncRNAs that are approximately 20–40 nucleotides in length. The primary transcription product of a miRNA gene, namely, pri-miRNA, is cleaved into precursor miRNA by RNase III Drosha in the nucleus and then transported to the cytoplasm, where it is further cleaved and matured by RNase III Dicer. Mature miRNAs target the 3’ untranslated region of mRNAs to regulate gene expression^27^. LncRNAs are ncRNAs that are at least 200 nucleotides in length. There are many lncRNAs, and they not only play auxiliary roles as intermediate carriers of genetic information but also perform various regulatory functions, such as genomic imprinting^28^, chromatin modification^29^, transcriptional activation^30^, and transcriptional interference^31^. Although a large number of studies on lncRNAs have been conducted in recent years, our understanding of them is limited, and the functions of most lncRNAs are still unknown; thus, we are interested in continuing to study these molecules^32^. CircRNAs are widely expressed in human cells, and pre-mRNAs form this unique ncRNA through reverse splicing. They do not have 5’ end caps and 3’ end poly(A) tails and exist as circular covalently closed structures. CircRNAs perform important regulatory functions, such as acting as competitive endogenous RNAs (ceRNAs)^33^.

The m6A methylation modification of ncRNAs with regulatory functions is an important part of epigenetic regulation. m6A modification affects various aspects of pathophysiological processes by changing the structure, biogenesis and function of ncRNAs, and it is crucial to the occurrence and progression of many diseases, especially cancer. This research hotspot has been reviewed in previous articles^34–38^. In recent years, many related studies have elucidated new mechanisms and provided new ideas. This review summarizes the mechanisms involved and discusses the importance of targeting m6A-modified ncRNAs in the treatment of cancer.

## Dysregulation of m6A writers in cancer

**Fig. 2:**
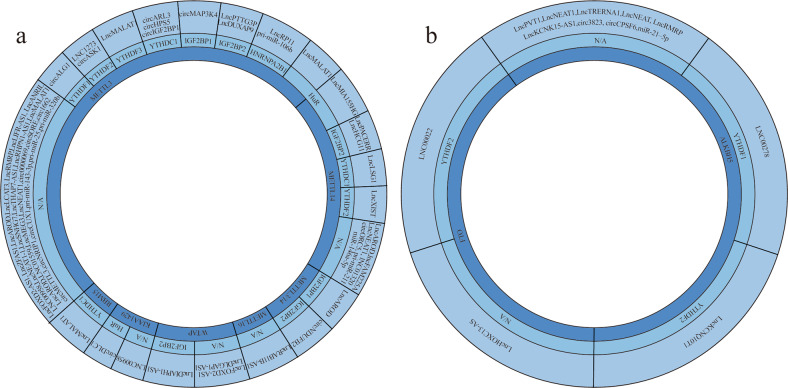
ncRNAs regulated by writers, erasers, and readers in different cancers. ncRNAs are modified by writer proteins and recognized by reader proteins (**a**). ncRNAs targeted by eraser proteins and recognized by reader proteins (**b**).

As mentioned above, the MTC, which primarily mediates m6A modification, consists of several proteins. In addition, some proteins, such as METTL16, function independently of the complex. A reader then recognizes and binds to the ncRNA at the m6A site. Figure 2a shows that ncRNAs are modified by dysregulated writers in tumors and that readers bind to these ncRNAs.

### METTL3

METTL3 is an S-adenosylmethionine (SAM)-binding protein. Its N-terminal extension includes a leading helix and a nuclear localization signal region, followed by a zinc finger domain (ZFD), a partly ordered linker and a C-terminal MTase domain (MTD). Among these domains, the MTD forms the smallest and most stable complex with the MTD and N-terminal extension of METTL14, which can bind to the cofactor substrate SAM or produce SAH^39^.

### Long noncoding RNA

METTL3 catalyzes the methylation of lncRNAs and increases their expression level by reducing RNA degradation and stabilizing RNA transcripts^40–55^. The increased stability of LncRMRP due to m6A modification activates the SMAD2/SMAD3 pathway by enhancing TGFBR1 transcription, ultimately accelerating non-small cell lung cancer (NSCLC) progression^42^. Conversely, after m6A methylation modification, some lncRNAs are downregulated due to reduced stability. Due to METTL3-mediated m6A modification, LncMEG3 expression is downregulated, its inhibitory effect on miR-544b is weakened, and the decreased expression of BTG2 promotes the progression of hepatocellular carcinoma (HCC)^56^.

m6A-modified lncRNAs sequester miRNAs, causing endogenous competition. Via METTL3 catalysis, the LncZFAS1 expression level remained unchanged, while the m6A level increased. LncZFAS1 sequesters miR-647 in an m6A-dependent manner to exert an overall cancer-promoting effect^57^.

Another important function of m6A-modified lncRNAs is binding to other molecules as scaffolds. By binding to CYCLINL1 at the m6A site, NEAT1-1 becomes a bridge that connects CYCLINL1 and CDK19 and then phosphorylates pol II Ser2 in the RUNX2 promoter, ultimately promoting the bone metastasis of prostate cancer (PC)^58^.

In addition, m6A modification increases the nuclear accumulation of lncRNAs. METTL3-mediated m6A modification leads to increased nuclear accumulation of LncRP11, and then, LncRP11 reduces the Siah1 and Fbxo45 levels and increases Zeb1 stability by forming the RP11-hnRNP A2B1-mRNA complex, ultimately playing an oncogenic role in colorectal cancer (CRC)^43^.

A recent study also found that METTL3 catalyzes m6A modification to promote lncRNA splicing. In pancreatic cancer (PAC), m6A-modified LncANRIL undergoes splicing with the participation of SRSF3. The spliceosome ANRIL-208 is increased and complexed with RING1b and EZH2 to mediate gemcitabine resistance by enhancing DNA HR repair^59^.

### Circular RNA

circRNAs, a type of ncRNA, usually lack the potential to be translated into proteins or peptides. However, after modification with m6A, they are able to be translated. m6A-methylated circMAP3K4, catalyzed by METTL3, can be translated into circMAP3K4-455aa with the help of IGF2BP1. This protein interacts with AIF to protect it from cleavage and reduce its nuclear distribution, ultimately leading to cisplatin resistance in HCC cells^60^.

METTL3 catalyzes circRNA methylation and increases its expression, mainly by reducing RNA degradation and stabilizing RNA transcripts^61–64^. METTL3-mediated m6A modification leads to the upregulation of circSORE, which sequesters miR-103a-2-5p and miR-660-3p. The Wnt/β-catenin pathway is activated, and HCC develops resistance to sorafenib^61^. Conversely, some m6A-modified circRNAs undergo endoribonucleolytic cleavage that is mediated by the reader, resulting in reduced stability. The protein ASK1-272a.a encoded by circASK1 competitively binds to Akt1, and the ASK1/JNK/p38 signaling pathway is activated to induce apoptosis and chemosensitivity in lung adenocarcinoma (LUAD)^65^.

m6A modification promotes circRNA back splicing and biosynthesis^66–68^. METTL3 overexpression mediates the m6A modification of circARL3 and promotes its biosynthesis. circARL3 acts as a ceRNA to absorb miR-1305, a molecule that inhibits target oncogenes, thereby promoting the growth of HBV + liver cancer cells^66^.

METTL3-mediated m6A modification promoted circRNA nuclear output. METTL3-mediated m6A modification leads to enhanced expression and cytoplasmic transport of circHPS5 and its increased ability to absorb miR-370 and upregulates HMGA2, ultimately accelerating HCC cell tumorigenesis^69^.

#### pre-microRNA

The biological significance of m6A modification in pre-microRNAs is mainly the promotion of their biological formation^70–72^. The current study mainly describes the following mechanisms: METTL3 promotes the splicing and maturation of miRNA precursors. METTL3-catalyzed m6A modification triggers the biogenesis of miR143-3p by promoting the splicing of pre-microRNA, leading to brain metastases in lung cancer^71^.

### METTL14

The catalytic domain of METTL14 does not contain SAM and exists in a closed conformation. METTL14 and METTL3 are connected by a loop near the active site of METTL3 to form a stable 1:1 heterodimer, which performs a more stable and powerful m6A catalytic function. The N-terminus of METTL14 contains a long helix that is approximately 500 amino acids long and interacts with METTL13, and an extension parallel to the N-terminal helix at the C-terminus achieves balance and functional coordination^73^. METTL14 increases the level of m6A methylation modification in lncRNAs and changes their stability^74–80^. METTL14 increases the levels of m6A-modified FAM225A and enhances its stability. FAM225A acts as a ceRNA to absorb miR-590-3p and miR-1275, eventually upregulating the target gene ITGB3 to promote nasopharyngeal carcinogenesis (NPC) cell tumorigenesis and metastasis^74^. When methylated by METTL14, lncRNAs act as molecular scaffolds and bind to m6A readers^81,82^. METTL14 catalyzes the m6A modification of LncPACERR. LncPACERR acts as a molecular scaffold to bind to IGF2BP2 and synergistically enhance the stability of KLF12 and C-MYC in TAMs^82^. In addition to IGF2BP2, m6A modifications catalyzed by METTL14 can also be recognized by other reader proteins, such as HuR, YTHDF1 and YTHDF2^78,79,81^.

METTL14 silencing leads to increased ncRNA expression in some tumors. Downregulation of METTL14 increases circORC5 expression in gastric cancer (GC). CircORC5 acts as a miR-30C-2-3p sponge and reverses the METTL14-induced upregulation of miR-30C-2-3p and downregulation of AKT1S1 and EIF4B, promoting GC cell growth and invasion^83^. The significance of METTL14-mediated m6A modification of pri-microRNAs is the increased maturation of its precursor^84,85^. When modified by METTL14, pri-microRNAs recruit DGCR8 and promote its maturation. METTL14 mediates the m6A modification of pri-miR-211, which in turn accelerates miR-211 biosynthesis through DGCR8 silencing. Increased miR-211 expression reduces cell proliferation in T-cell lymphoblastic lymphoma (T-LBL) by targeting the miR-211/TCF12 axis^85^.

### METTL3/14

METTL3/14 catalyzes the m6A modification of ncRNAs and stabilizes their transcripts. After modification, LncAROD exhibits enhanced stability and directly binds to SRSF3 to induce the switch from PKM to PKM2. As a ceRNA that inhibits miR-145-5p, LncAROD enhances glycolysis, cell proliferation, migration, invasion and chemical resistance of HCC cells^76^. When METTL3/14 cooperate to catalyze m6A methylation modification, ncRNAs act as molecular scaffolds to promote the formation of various complexes. For instance, circNDUFB2 forms a ternary complex with TRIM25 and IGF2BPs to promote the ubiquitination and degradation of IGF2BPs, which are tumor-promoting factors^86^.

### WTAP

WTAP is a splicing regulator that contains an extended N-terminal helical coil region and an unstructured C-terminal portion. The N-terminal portion of WTAP interacts with the N-terminal leader helix (LH) of METTL3, allowing METTL3/14 to localize to the nuclear speckle and play an important role in RNA m6A methylation^87^. WTAP promotes the methylation modification of ncRNAs and favors their stability^88–90^. WTAP enhances the stability of transcripts by promoting the m6A modification of LncFOXD2-AS1, and FOXD2-AS1 binds to its target FOXM1 mRNA to form a complex that promotes the stability of the latter and ultimately promotes the occurrence of osteosarcoma (OS)^89^. WTAP binds to the m6A site of LncDLGAP1-AS1 to improve its stability, and DLGAP1-AS1 sponges miR-299-3p to reverse the inhibition of WTAP mRNA, forming a regulatory loop and promoting adriamycin (ADR) resistance in breast cancer (BRC)^90^.

### KIAA1429

KIAA1429, the largest factor related to m6A, is localized to NSs and has been shown to recruit METTL3, METTL14 and WTAP to guide regionally selective methylation. KIAA1429-catalyzed m6A modification typically occurs near 3’-UTRs and stop codons. Similar to METTL3 and METTL14, KIAA1429-mediated m6A methylation may cause changes in the expression of some ncRNAs. In HCC, KIAA1429 catalyzes the m6A methylation modification of circDLC1, reduces its expression level, and promotes tumor progression by altering the circDLC1-HuR-MMP1 axis^91^.

### RBM15

RBM15 recruits MTC to its target transcript by directly binding to U-rich sequences in RNA^92^. RBM15 specifically targets ncRNA secondary structures, such as the A-repeat region of LncXIST. There is a conserved AUCG tetraloop hairpin in the A repeat region of LncXIST. RBM15 recruits the write complex to XIST for m6A modification by binding to the A-repeat, and the YTH domain recognizes the m6A site in the A-repeat (m6A) UCG tetraloop. The (m6A) UCG tetraloop of the XIST A-repeat hairpin RNA is bound by an arc-like surface of the YTH domain^93^. In esophageal squamous cell carcinoma (ESCC), RBM15 interacts with METTL3 in a WTAP-dependent manner to deposit m6A on LncMALAT1. LncMALAT1 binds to YTHDC1 as a molecular scaffold to maintain its nuclear localization and related oncogene expression, ultimately promoting metastasis of cancer cells^94^.

Abnormal m6A modification of ncRNAs that is regulated by writer proteins affects tumor development. We summarize the role of methyltransferase and its target ncRNAs in various cancers (Table 1).

**Table 1:** The role of methyltransferases and their regulated ncRNAs in various cancers.

Writer	Role in cancer	Type	Target ncRNAs	Mechanism
METTL3	Oncogene	CC	LncFOXD2-AS1^50^	Stability
			circ0000069^63^	Stability
			LncZFAS1^57^	Adsorb microRNAs
		HCC	LncAROD^49^	Stability
			LNC1273^55^	Stability
			circMAP3K4^60^	Translation
			circSORE^61^	Stability
			circARL3^66^	Circularization
			circHPS5^69^	Cytoplasmic output
		CRC	LncPTTG3P^41^	Stability
			LncRP11^43^	Nuclear accumulation
			circALG1^64^	Stability
			circ1662^67^	N/A
		LUAD	LncLCAT3^47^	Stability
			pri- miR-143-3p^71^	Maturation
			pri-miR-106b^133^	Maturation
		NSCLC	LncRMRP^41^	Stability
			circIGF2BP3^67^	Circularization
		PAC	LncLIFR-AS1^51^	Stability
			LncANRIL^58^	Splicing
			pri-miR-25^70^	Maturation
		BRC	LNC00958^46^	Stability
			LncMALAT1^122^	Stability
		PC	LncNEAT1-1^58^	Scaffold
			LncSNHG7^54^	Stability
		GC	LncTHAP7-AS1^48^	Stability
		EOC	LncRHPN1-AS1^44^	Stability
		TET	LncMALAT1^51^	Stability
		RCC	LncDUXAP9^45^	Stability
		HNSCC	LncAROD^76^	Stability
		HPSCC	circCUX1^62^	Stability
		ESCC	pri-miR-320b^72^	Maturation
	Tumor suppressor	LUAD	circASK1^65^	Stability
		GBM	LncMALAT1^53^	Stability
		HCC	LncMEG3^56^	N/A
METTL14	Oncogene	HNSCC	LncAROD^76^	Stability
		NPC	LncFAM225A^74^	Stability
		HCC	LncMIR155HG^79^	Stability
		GC	LNC01320^75^	Stability
			circORC5^83^	N/A
		TLBL	pri-miR-211^85^	Maturation
		BRC	miR-146a-5p^84^	Reshape the miRNA profile
		CRC	LncXIST^78^	Stability
		PAC	LncPACERR^82^	Scaffold
		RCC	LncLSG1^81^	Scaffold
	Tumor suppressor	RCC	LncNEAT1^80^	Stability
	Tumor suppressor	LUAD	LncHCG11^77^	Stability
METTL16	Tumor suppressor	HCC	LncRAB11B-AS1^97^	Stability
METTL3/14	Oncogene	HCC	LncAROD^49^	Stability
	Oncogene	NSCLC	circNDUFB2^86^	Scaffold
WTAP	Oncogene	NPC	LncDIAPH1-AS1^88^	Stability
	Oncogene	OS	LncFOXD2-AS1^50^	Stability
	Oncogene	BRC	LncDLGAP1-AS1^90^	Stability
KIAA1429	Oncogene	GC	LNC00958^149^	Stability
	Tumor suppressor	HCC	circDLC1^91^	N/A
RBM15	Oncogene	ESCC	LncMALAT1^94^	Scaffold

*CC* cervical cancer, *HCC* hepatocellular carcinoma, *CRC* colorectal cancer, *LUAD* lung adenocarcinoma, *NSCLC* non-small cell lung cancer, *PAC* pancreatic cancer, *BRC* breast cancer, *PC* prostate cancer, *GC* gastric cancer, *EOC* epithelial ovarian cancer, *TET* thymic epithelial tumor, *RCC* renal cell carcinoma, *HNSCC* head and neck squamous cell carcinoma, *HPSCC* hypopharyngeal squamous cell carcinoma, *ESCC* esophageal squamous cell carcinoma, *GBM* glioblastoma multiforme, *NPC* nasopharyngeal carcinogenesis, *TLBL* T-cell lymphoblastic lymphoma.

### METTL16

The conserved core of the SAM methyltransferase METTL16 consists of β-sheets, α-helices and 310(η) helices. The order of the 7-strand β-sheets is 3214576, located between the α and 310 (η) helical clusters. SAM and RNA binding sites are created within the N-terminal and C-terminal segments of the β-sheet, respectively^95^. In some ncRNAs, such as MALAT1, the U-rich internal loop at the 3’ end of MALAT1 associates with a downstream genomically encoded A-rich stretch to form a bipartite triple helix composed of canonical triples: nine U•A-U, one C•G-C, and a C-G doublet. The triple helix structure stabilizes MALAT1 and allows it to accumulate in cells^96^. METTL16 recognizes the RNA triple helix at the 3’ end of MALAT1^95^. Similar to METTL3 and METTL14, METTL16 also affects the stability of ncRNA by inducing the catalytic modification of ncRNAs with m6A. METTL16 catalyzes the m6A modification of LncRAB11B-AS1 to reduce its stability. When LncRAB11B-AS1 expression is downregulated, the malignant phenotype of HCC cells is inhibited^97^.

## Dysregulation of m6A erasers in cancer

To date, two types of erasers have been identified, namely, FTO and ALKBH5. Although there are few types of eraser proteins, their dysfunction has been observed in a variety of tumors. In light of their important role in cancer, we list demethylated ncRNAs (Fig. 2b) and explain their mechanisms of action.

### FTO

FTO was the first RNA demethylase to be discovered, and its dysregulation plays tumor-promoting or tumor-suppressing roles^13^. There are two pairs of positively charged residues that bind to oligonucleotide-like pincers in two loops near the oligonucleotide-binding region of FTO. A hydrophobic pocket is formed in the inner chain of the catalytic pocket of FTO, and the N6 methyl group is stably bound in this pocket for oxidation to Fe(II) and α-KG^98^. FTO erases the m6A methylation modification of ncRNAs, ultimately inhibiting the degradation of ncRNAs through m6A readers. In ESCC, FTO removes the m6A modification of LNC00022, leading to the inhibition of LNC00022 degradation via the m6A reader YTHDF2 and promoting tumor proliferation by accelerating P21 ubiquitination and degradation^99^. FTO increases the stability of ncRNAs by reducing methylation modification. When targeted by FTO, LncHOXC13-AS is upregulated and interacts with CBP to induce FZD6 upregulation, promoting Wnt/β-catenin activation and ultimately leading to increased cervical cancer (CC) invasiveness^100^.

### ALKBH5

Similar to all eukaryotic ALKBH family members, the basic scaffold of ALKBH5 consists of eight antiparallel β-sheets, and the secondary structure motifs are essential for the catalytic function of ALKBH5. For example, motif 1 is associated with the activity of the ALKBH5 enzyme, and its substrate specificity is conferred by several motifs, including important active site coordinating residues HxD. H motif and motif 2, which specifically bind to the m6A single-chain substrate body^101^. ALKBH5 increases the stability of ncRNAs^102–104^. Under hypoxic conditions, the stability of LncNEAT1 is increased because its m6A modification is eliminated by ALKBH5, and the posttranscriptional repressor SFPQ is relocated from its position in the CXCL8 promoter to paraspeckles, ultimately upregulating CXCL8/IL8 and promoting glioblastoma multiforme (GBM) progression^103^. Conversely, ALKBH5 also reduces the stability of ncRNAs^105,106^. In PAC, downregulation of ALKBH5 allows demethylation and decreased expression of LncKCNK15-AS1, which inhibits epithelial–mesenchymal transition, resulting in tumor invasion and metastasis^105^. ALKBH5 upregulates the level of catalyzed ncRNAs^107–110^. ALKBH5 upregulates circCPSF6 through demethylation, and circCPSF6 competitively interacts with PCBP2 to stabilize YAP1 mRNA and promote the malignant phenotype of HCC^110^. ALKBH5 upregulates LncNEAT1 expression through demethylation, leading to the overexpression of a subunit of the polycomb repressive complex EZH2 and ultimately promoting the malignant phenotype of GC^107^. ncRNAs modified by ALKBH5 act as molecular scaffolds. ALKBH5 catalyzes the demethylation of LncTRERNA1 and upregulates its expression. LncTRERNA1 acts as a molecular scaffold to recruit EZH2 and silence its expression through H3K27me3 modification of the promoter region of P21, which is a cyclin-dependent kinase inhibitor, thus promoting cell proliferation and cell cycle progression in diffuse large B-cell lymphoma (DLBCL)^111^. ncRNA molecules that are targeted by ALKBH5 have silencing potential. ALKBH5 deficiency increases the level of m6A modification of miR-21-5p and increases the expression level of miR-21-5p target genes, such as MAPK10, PTEN, SMAD7, PDCD4 and SOX7, in NSCLC^112^.

The dysregulation of ncRNA m6A modification catalyzed by eraser proteins plays an important role in tumorigenesis and progression. We summarize the role of demethylases and their target ncRNAs in various cancers (Table 2).

**Table 2:** The role of demethylases and their regulated ncRNAs in various cancers.

Eraser	Role in cancer	Type	Target ncRNAs	Mechanism
FTO	Oncogene	ESCC	LNC00022^99^	Stability
		CC	LncHOXC13-AS^100^	Stability
ALKBH5	Oncogene	OS	LncPVT1^104^	Stability
		GBM	LncNEAT1^103^	Stability
		LSCC	LncKCNQ1OT1^117^	Stability
		DLBCL	LncTRERNA1^111^	Scaffold
		CRC	circ3823^102^	Stability
		HCC	circCPSF6^110^	Stability
		NSCLC	miR-21–5p^112^	Silencing potency
		GC	LncNEAT1^107^	N/A
		CRC	LncNEAT1^109^	N/A
		LUAD	LncRMRP^108^	N/A
	Tumor suppressor	ESCC	LNC00278^106^	Translation
	Tumor suppressor	PAC	LncKCNK15-AS1^105^	N/A

*ESCC* esophageal squamous cell carcinoma, *CC* cervical cancer, *OS* osteosarcoma, *GBM* glioblastoma multiforme, *LSCC* laryngeal squamous cell carcinoma, *DLBCL* diffuse large B-cell lymphoma, *CRC* colorectal cancer, *HCC* hepatocellular carcinoma, *NSCLC* non-small cell lung cancer, *GC* gastric cancer, *LUAD* lung adenocarcinoma, *PAC* pancreatic cancer.

**Fig. 3:**
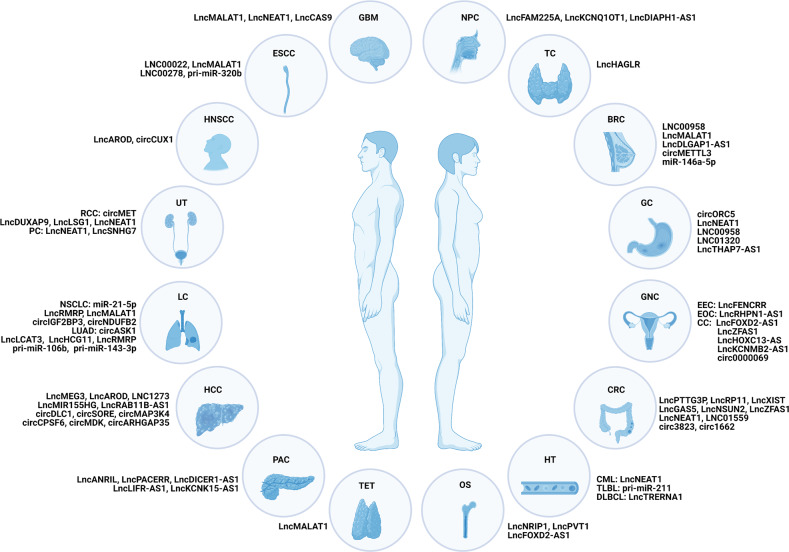
Dysregulation of m6A-modified ncRNAs in human cancer. GBM glioblastoma multiforme, ESCC esophageal squamous cell carcinoma, LSCC laryngeal squamous cell carcinoma, HNSCC head and neck squamous cell carcinoma, UT urinary tract, RCC renal cell carcinoma, BC bladder cancer, PC prostate cancer, LC lung cancer, NSCLC non-small cell lung cancer, LUAD lung adenocarcinoma, HCC hepatocellular carcinoma, PAC pancreatic cancer, TET thymic epithelial tumor, OS osteosarcoma, HT hematological system, CML chronic myeloid leukemia, TLBL T-cell lymphoblastic lymphoma, DLBCL diffuse large B-cell lymphoma, CRC colorectal cancer, GNC genital system, EEC endometrioid endometrial carcinoma, EOC epithelial ovarian cancer, CC cervical cancer, GC gastric cancer, BRC breast cancer, TC thyroid cancer, NPC nasopharyngeal carcinogenesis.

Thus far, it is not difficult to find that dysregulated m6A modification of ncRNAs, including increased m6A modification caused by methyltransferases and decreased m6A modification by demethylases, affects the development of various cancers. These changes have been observed in more than 20 tumors (Fig. 3).

## Dysregulation of m6A readers in cancer

### YTHDF1

The YTH domain of YTHDF1 consists of 5 α helices (α0-α4), 6 parallel β chains (β1-β6), and a 3_10_ helix following the β5 chain. In this YTH domain, the rings between β4β5 and β1α1β2 at the C-terminus and α2 at the N-terminus form the m6A-binding pocket. The aromatic cage composed of Trp411, Trp465 and Trp470 specifically recognizes m6A-modified sites^113^. YTHDF1 is the most versatile and robust of all reader proteins; it recognizes G (m6A) C and A (m6A) C RNA sequences without sequence selectivity^114^. YTHDF1 binds to m6A sites on ncRNAs and recruits translation machinery to increase translation efficiency. Yin Yang 1 (YY1)-binding micropeptide is translated by m6A-modified LNC00278 with the help of YTHDF1, which blocks the interaction between YY1 and the androgen receptor AR, reduces the expression of eEF2K through the AR signaling pathway, increases the level of caspase-3, and causes ESCC cells to undergo apoptosis. ALKBH5-mediated demethylation in patients who smoke inhibits this process and ultimately mediates the malignant phenotype of ESCC^106^.

### YTHDF2

The YTH region of YTHDF2 has a spherical shape overall, with 4 β-strands (β1-β4) in the center, 4 α-helices (α1-α4) around it, and flanking regions on each side. The YTH surface has a structure that is rich in basic residues, in which the K416 residue on the β1 strand and the R527 residue on the linker helix α3α4 are essential for binding m6A methyl groups. In the nearby hydrophobic pocket, residues W432 and W486 are involved in the specific recognition of m6A modifications^115^. According to high-throughput sequencing, YTHDF1 and YTHDF2 have no sequence preference and recognize the m6A site equally^116^. YTHDF2 binds to m6A-modified sites in lncRNAs to promote their transcriptional stability. In laryngeal squamous cell carcinoma (LSCC), decreased ALKBH5 levels lead to increases in m6A modification of LncKCNQ1OT1 and enhanced LncKCNQ1OT1 stability mediated by YTHDF2. LncKCNQ1OT1 directly binds to HOXA9 to further regulate the proliferation, invasion and metastasis of LSCC cells^117^. When m6A is recognized by YTHDF2, ncRNAs are degraded^65,78,99,118^. METTL14 induces m6A methylation modification of LncXIST and reduces XIST expression through YTHDF2-dependent RNA degradation, which plays an important role in CRC progression^78^. Similarly, FTO decreases the m6A modification of LNC00022, and the weakened binding of YTHDF2 accelerates the ubiquitination and degradation of the P21 protein by inhibiting the degradation of LNC00022, thus promoting the proliferation of ESCC^99^.

### YTHDF3

YTHDF3, the third member of the YTH family, functions as a partner of YTHDF1 and YTHDF2 to promote translation by recognizing m6A sites and affecting the cytoplasmic metabolism of RNAs^119,120^. By recognizing m6A sites, YTHDF3 changes the stability of ncRNAs^118,121,122^. LncDICER1-AS1 promotes the transcription of its sense gene DICER1 by recruiting the YY1 transcription factor to the DICER1 promoter. DICER1 promotes the maturation of miR-5586-5p, thereby inhibiting the expression of the glycolytic genes LDHA, HK2, PGK1 and SLC2A1. YTHDF3 binds to the m6A site of LncDICER1-AS1 to promote its degradation, which ultimately enhances the glycolysis of PAC cells^118^. YTHDF3 recognizes the m6A site of LncGAS5 and promotes its ubiquitin-mediated degradation. After the content of LncGAS5 decreases, its ability to promote the degradation of YAP decreases, and the level of YAP increases. YTHDF3 is the target of YAP, and its level increases with increasing YAP levels. The three form a regulatory loop that promotes the progression of CRC^105^.

There are strong debates regarding whether DF1/DF2 have distinct roles or redundant roles. Previously, different YTHDF paralogs were thought to be bound to different m6A sites to perform different functions^24,119,123,124^. However, with advances in research, new studies have proposed that the YTH domain and all m6A sites are equally bound and that the three DF paralogs act redundantly to mediate mRNA degradation and cellular differentiation^125–127^. These studies reveal a unified model of m6A function in which all m6A-modified mRNAs are subjected to the combined action of YTHDF proteins in proportion to the number of m6A sites^126^.

### YTHDC1

The YTH domain consists of 5 α-helices (α0-α4), 6 β-chains (β1-β6) and 3_10_ helices. The m6A binding pocket consists of a β1 residue, a ring between α1 and β1, α1, β1 and a partial ring connecting the β4 and β5 chains, while the aromatic pocket that recognizes m6A modification sites consists of W377, W428 and L439^128^. YTHDC1 regulates RNA metabolic processes in a m6A-dependent manner^16,129^. YTHDC1 locates oncogenes in nuclear spots by recognizing the m6A site in ncRNAs, amplifying the expression of oncogenes. The m6A site in MALAT1 acts as a scaffold for recruiting YTHDC1 to NSs and promotes the upregulation of key oncogenes in ESCC^94^. YTHDC1 binds to the m6A site of ncRNAs to promote their reverse splicing and biosynthesis^66,68^. METTL3 overexpression mediates the m6A modification of circARL3, and YTHDC1 binds to the m6A site and promotes its biosynthesis. circARL3 acts as a ceRNA to absorb miR-1305 and causes downregulation of WNT2, UBE2T, MDM2, TGF-β2 and POLR3G, thereby promoting the growth of HBV + HCC cells^66^. Another example is YTHDC1, which recognizes the site of METTL3-mediated m6A modification in circIGF2BP3 and promotes circIGF2BP3 cyclization. circIGF2BP3 acts as a ceRNA to absorb miR-328-3p and miR-3173-5p and upregulates the expression of PKP3, thereby destroying tumor immunity in NSCLC^68^. YTHDC1 promotes the nuclear output of ncRNA^69,130^. YTHDC1 promotes the nuclear output of ncRNAs. With the help of YTHDC1, m6A-modified circMET is transported to the cytoplasm, where it promotes the growth of NONO-TFE3 tRCC by enhancing CDKN2A mRNA attenuation, competitively absorbing miR1197 and blocking SMAD3 mRNA expression^130^. METTL3-mediated m6A modification increases the expression of circHPS5, while YTHDC1 promotes the transport of circHPS5 to the cytoplasm. These two act together to increase the ability of circHPS5 to function as a ceRNA to absorb miR-370, upregulate HMGA2, and promote the progression of HCC^69^.

### YTHDC2

YTHDC2 affects tumor progression by regulating RNA metabolism. YTHDC2 affects tumor progression by regulating RNA metabolism. YTHDC2 binds to m6A-modified mRNA and promotes its degradation. m6A-modified SLC7A11 mRNA binds to YTHDC2, which enhances the degradation of YTHDC2 and reduces cystine uptake, thereby blocking the downstream antioxidant program and ultimately suppressing the progression of LUAD^131^. In addition, YTHDC2 binds to mRNA to promote its translation initiation. YTHDC2 binds to IGF1R mRNA and promotes its translation initiation to activate the IGF1R-AKT/S6 axis, which ultimately leads to NPC radioresistance^132^. However, no study has shown the role of YTHDC2 in ncRNA m6A modification.

### IGF2BP1

IGF2BP1, the first IGF2BP family member to be described, consists of six typical binding domains, including four C-terminal K homology (KH) domains that promote RNA binding and two N-terminal RNA recognition domains (RRMs) that promote the stability of IGF2BP-RNA complexes^133^. IGF2BP1 binds to m6A sites in ncRNAs, increasing their stability. IGF2BP1 recognizes the m6A modification site in circMDK to increase the stability of its transcripts. circMDK sponges miR-346, and miR-874-3p upregulates autophagy-related 16-like 1 (ATG16L1), thereby activating the PI3K/AKT/mTOR signaling pathway and promoting HCC cell proliferation, migration and invasion^134^. METTL3 and IGF2BP1 act together to increase the stability of LncAROD through m6A modification. Elevated LncAROD increases the levels of pyruvate kinase isoform M2 (PKM2) in hypoxic environments or HIF1α and promotes HCC progression^49^.

### IGF2BP2

IGF2BP2 is widely expressed in a variety of tissues and is an independent prognostic factor for various cancer types; it functions by recognizing the m6A sites of different types of RNAs, such as miRNAs, mRNAs and LncRNAs, that are involved in the occurrence and growth of cancer^135^. IGF2BP2 increases the stability of ncRNAs^41,45,77,136–140^. For example, IGF2BP2 recognizes and stabilizes LncDANCR harboring m6A methylation, and then, these molecules work together to promote the progression of PC^137^. IGF2BP2 also contributes to the formation of multiplex complexes. The m6A methylation-modified circNDUFB2 functions as a scaffold that forms a ternary complex with TRIM25 and IGF2BPs to promote the ubiquitination and degradation of the NSCLC-promoting factor IGF2BPs^86^. When circNSUN2 undergoes m6A modification by METTL3, its transport to the cytoplasm increases with the help of YTHDF2. CircNSUN2 enters the cytoplasm to form a circNSUN2/IGF2BP2/HMGA2 RNA protein ternary complex, increasing the stability of HMG mRNA and enhancing CRC malignancy^141^.

### IGF2BP3

Similar to IGF2BP1, IGF2BP3 is expressed in only a few tissues and cells of healthy adults but is resynthesized or substantially upregulated in various tumors. Therefore, to some extent, they can be characterized as oncofetal genes^142^. IGF2BP3 recognizes and binds to the m6A site of ncRNAs to improve their stability. IGF2BP3 increases the expression of LncKCNMB2-AS1 by binding to the m6A methylation modification site. Upregulated LncKCNMB2-AS1 acts as a competing endogenous RNA of miR-130b-5p and miR-4294 and ultimately facilitates CC progression^143^.

### hnRNPs

Heterogeneous nuclear ribonucleoproteins (hnRNPs) are a class of RNA-binding proteins (RBPs). There are four unique RNA-binding domains (RBDs) in hnRNP proteins: RNA recognition mods (RRM), quasi-RRM, glycine rich domains that constitute RGG boxes, and KH domains. RRM is the most common RBD, and it includes two conserved RNP1 octamer and RNP2 hexacomer sequences and four β-sheets and two α-helices (βαβαβ) that are critical for RNA binding specificity. hnRNPs play an important role in many aspects of nucleic acid metabolism^144^. hnRNP A2B1 promotes the maturation of miR-106b-5p by reading the m6A site in pri-miR-106b, thereby reducing the level of secreted frizzled-related protein 2 (SFRP2) to activate the Wnt/β-catenin signaling pathway and promote LUAD tumor occurrence^145^. hnRNP A2B1 binds to the m6A site in LncRP11 to increase its nuclear accumulation and recruit Siah1 and Fbxo45 mRNA. Enhanced mRNA degradation of two E3 ligases, Siah1 and Fbxo45, prevents Zeb1 proteasome degradation and ultimately promotes the CRC malignant phenotype^43^.

### HuR

HuR is an RNA-binding protein that recognizes U/AU-rich elements in different RNAs through two RNA recognition motifs, namely, RRM1 and RRM2. The RRM domain adopts a β1-α1-β2-β3-α2-β4 topology with two α-helices stacked in an antiparallel four-stranded β-sheet. Residues located at conserved positions on β strands 1 and 3 are critical for RN binding. HuR tightly regulates target RNA fate at the posttranscriptional level^146^. HuR binds to the m6A sites of ncRNAs to stabilize their transcripts^53,79^. Lipopolysaccharide (LPS) of intestinal bacteria upregulates METTL14, promotes LncMIR155HG methylation and stabilizes it in a HuR-dependent manner. LncMIR155HG acts as a ceRNA to induce PD-L1 expression through the miR-223/STAT1 axis and plays an important role in immune escape in HCC^79^.

### EIF3

Translation initiation in eukaryotes relies on a number of eukaryotic initiation factors (eIFs) that stimulate the recruitment of initiating tRNA, Met-TRNAiMet, and mRNA to the 40S ribosomal subunit and subsequently scan the mRNA for the AUG initiation codon. EIF3, one of the first initiating factors to be discovered in the 1970s, organizes a network of interactions among several eIFs. Studies have found that circRNAs, as ncRNAs, do not have the ability to be translated into proteins or peptides, but when they undergo m6A modification and EIF3 binds to the m6A site, some ncRNAs can be translated. When EIF3 binds to the m6A modification site in the initiation codon of circARHGAP35, a truncated protein that contains four FF domains and lacks the Rho GAP domain is produced, and this protein promotes tumor progression by interacting with the TFII-I protein in the nucleus^19^.

**Fig. 4:**
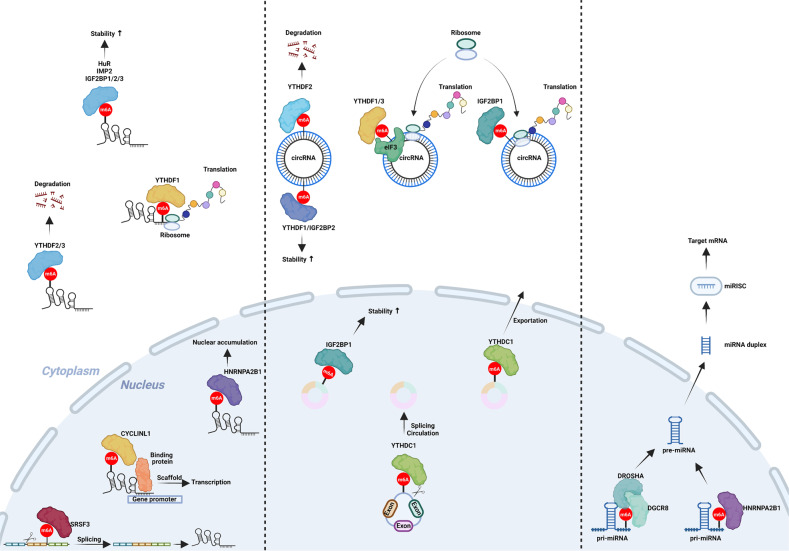
Functions of m6A modifications in noncoding RNAs. The functions of m6A modifications in circRNAs (left) include reverse shearing, changes in stability, and enhanced cytoplasmic transport; these circRNAs are also able to be translated. The functions of m6A modification in lncRNAs (middle) include splicing, acting as molecular scaffolds, nuclear accumulation, and stability changes, and these lncRNAs can also be translated. The functions of m6A modifications (right) are mainly to promote their maturation.

After the m6A modifications of ncRNAs are installed by writers or removed by erasers, the ability of reader proteins to bind to these molecules changes, and the metabolic fate of these ncRNAs is determined by different mechanisms (Fig. 4). We summarize the roles of reader proteins and their target ncRNAs in a variety of tumors (Table 3).

**Table 3:** Dysregulation of m6A-binding proteins in cancer.

Reader	Role in cancer	Type	Target ncRNAs	Mechanism
YTHDF1	Oncogene	CRC	circALG1^64^	Stability
	Tumor suppressor	ESCC	LNC00278^106^	Translation
YTHDF2	Oncogene	CRC	lncXIST^78^	Stability
		ESCC	LNC00022^99^	Stability
		LSCC	LncKCNQ1OT1^117^	Stability
		HCC	LNC1273^55^	Stability
			circCPSF6^110^	Stability
	Tumor suppressor	EEC	LncFENDRR^155^	Stability
		LUAD	circASK1^65^	Stability
YTHDF3	Oncogene	BRC	LncMALAT1^122^	Stability
	Tumor suppressor	CRC	LncGAS5^121^	Stability
		PAC	LncDICER1-AS1^118^	Stability
YTHDC1	Oncogene	ESCC	LncMALAT1^94^	Scaffold
		HCC	circARL3^66^	Circularization
			circHPS5^69^	Cytoplasmic output
		RCC	LncLSG1^81^	Scaffold
			circMET^130^	Cytoplasmic output
		NSCLC	circIGF2BP3^68^	Circularization
IGF2BP1	Oncogene	HCC	LncAROD^49^	Stability
			circMDK^134^	Stability
			circMAP3K4^60^	Translation
IGF2BP2	Oncogene	CRC	LncPTTG3P^41^	Stability
			circNSUN2^141^	Cytoplasmic export
			LncZFAS1^139^	Stability
		GBM	lncCASC9^136^	Stability
		NPC	LncDIAPH1-AS1^88^	Stability
		RCC	LncDUXAP9^45^	Stability
		PAC	LncDANCR^137^	Stability
			LncPACERR^82^	Scaffold
		TC	LncHAGLR^138^	Stability
		NSCLC	LncMALAT1^140^	Stability
	Tumor suppressor	NSCLC	circNDUFB2^86^	Scaffold
		LUAD	lncHCG11^77^	Stability
IGF2BP3	Oncogene	CC	lncKCNMB2-AS1^143^	Stability
HNRNPA2B1	Oncogene	LUAD	pri-miR-106b^145^	Maturation
		CRC	LncRP11^43^	Nuclear accumulation
EIF3	Oncogene	HCC	circARHGAP35^19^	Translation
HuR	Oncogene	HCC	LncMIR155HG^79^	Stability
		GBM	LncMALAT1^53^	Stability
	Tumor suppressor	HCC	circDLC1^91^	N/A

*CRC* colorectal cancer, *ESCC* esophageal squamous cell carcinoma, *LSCC* laryngeal squamous cell carcinoma, *HCC* hepatocellular carcinoma, *EEC* endometrioid endometrial carcinoma, *LUAD* lung adenocarcinoma, *BRC* breast cancer, *PAC* pancreatic cancer, *RCC* renal cell carcinoma, *NSCLC* head and neck squamous cell carcinoma, *GBM* glioblastoma multiforme, *NPC* nasopharyngeal carcinogenesis, *TC* thyroid cancer, *CC* cervical cancer.

## The clinical potential of ncRNAs and m6A modifiers

With ncRNA methylation playing such an important role in tumor progression, m6A-modified ncRNAs and various m6A effectors, including writers, erasers, and readers, may be potential tumor markers. Chen et al. analyzed the circRNA expression profiles of 6 pairs of CRC tissues and adjacent nontumor tissues by high-throughput sequencing and found that circ1662 expression was increased and correlated with poor prognosis and depth of tumor invasion in CRC. Moreover, the level of METTL3 in CRC tissues was higher than the level of METTL3 in adjacent tissues and predicted poor prognosis. Thus, METTL3 and its modified circ1662 have the potential to serve as biomarkers to judge the prognosis of CRC^67^. Shen et al., showed through high-throughput methylated RNA immunoprecipitation sequencing of RCC cells that LncLSG1 was upregulated after m6A modification, which was closely related to tumor metastasis. Meanwhile, significantly high expression of METTL14 in RCC tissues resulted in tumor metastasis and poor OS and DFS. In conclusion, METTL14 and its modified lncRNA LncLSG1 can be considered potential markers to predict RCC metastasis^81^. Decreased expression of circDLC1, which is regulated by KIAA1429, is associated with more advanced HCC stages^91^. In CRC, high expression of m6A-modified LNC00460 is associated with lower 5-year overall survival and disease-free survival rates^147^. Increased LncTHAP7-AS1 expression after m6A modification is associated with poor prognosis of GC^48^. The expression of m6A-modified LncRHPN1-AS1 is an important indicator for assessing the prognosis of epithelial EOC^44^. Notably, the same m6A-modified ncRNA can play a tumor-promoting role in many tumors, suggesting that this molecule may be a potential tumor marker. For example, increased expression of LncMALAT1 affects the malignant phenotype of various tumors. The increased LncMALAT1 expression observed in TC stimulates the proliferation, migration and invasion of TC cells by competitively binding to miR-204, upregulating IGF2BP2 and enhancing MYC expression^148^. In ESCC, LncMALAT1 acts as a molecular scaffold to bind to YTHDC1, which is crucial for the maintenance of NSs and the expression of related oncogenes, thereby promoting cancer cell metastasis^94^. In TET, LncMALAT1 induces high expression of c-MYC protein to promote proliferation and metastasis^51^. Increased stability of LncMALAT1 in GBM activates the NF-κB signaling pathway to promote tumor growth, and this process is positively associated with higher malignant grade and poorer prognosis^53^. Moreover, activation of the MALAT1/miR-26b/HMGA2 axis in BC leads to epithelial-mesenchymal transition and enhanced tumor cell invasion^122^. LNC00958 also plays a role in a variety of tumors. In BC, LNC00958 upregulates YY1, which functions as a ceRNA for miR-378a-3p, promoting tumor progression^46^. In GC, LNC00958 positively regulates aerobic glycolysis by enhancing the transcriptional stability of GLUT1 mRNA, promoting tumor progression and reducing the survival rate of patients^149^. LNC00958 sponges miR3619-5p in HCC, leading to the upregulation of hepatoma-derived growth factor (HDGF) and thereby promoting HCC adipogenesis and progression^40^. LncNEAT1 also plays a role in the initiation and progression of various tumors. LncNEAT1 binds to CYCLINL1 at m6A sites and acts as a bridge between CYCLINL1 and CDK19, promoting the bone metastases of PC by phosphorylating Pol II Ser2 in the RUNX2 promoter^58^. The increase in LncNEAT1 expression relocates the posttranscriptional repressor SFPQ from its position in the CXCL8 promoter to paraspeckles, which ultimately upregulates CXCL8/IL8 and promotes the progression of GBM^103^. Upregulated LncNEAT1 expression causes the overexpression of a subunit of the polycomb repressive complex EZH2, which ultimately promotes the malignant phenotype of GC^107^.

RNA-modifying effectors may be targets for therapeutic intervention. m6A has been studied for decades, but earlier studies focused on DNA methylation. Therefore, both azacytidine and decitabine, which have been approved for the clinical treatment of tumors, are DNA methyltransferase inhibitors^150,151^. Although RNA methylation was more recently discovered, a number of small molecules that inhibit the RNA demethylation enzyme FTO have been identified^152–154^. For example, the natural compound rhein has been shown to competitively bind to FTO to inhibit its effects on its ssRNA or ssDNA substrates^152^. An FTO-specific inhibitor, meclofenamic acid (MA), inhibits the demethylation of m6A-modified ssRNA or ssDNA by inhibiting FTO^153^. The efficacy of anti-PD-1 therapy in METTL3 or METTL14 KO mice as well as ALKBH5 KO mice is increased.

**Fig. 5:**
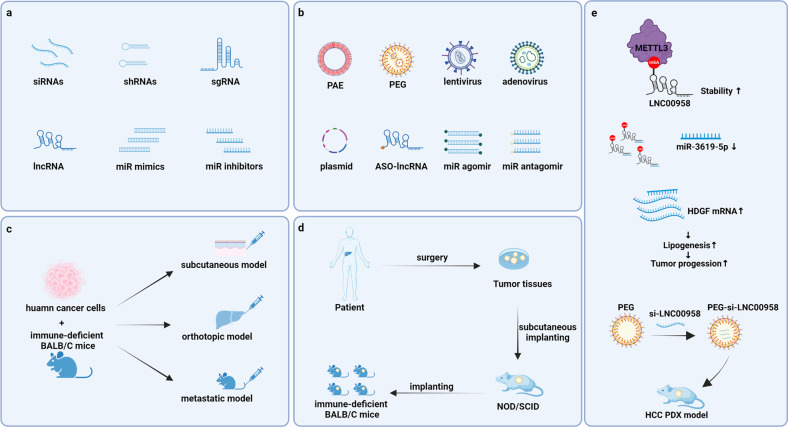
ncRNA-based therapeutics and delivery systems in a mouse model. Several ncRNA-based therapeutics exist, including siRNAs, shRNAs, sgRNAs, therapeutic lncRNAs, miRNA mimics and miR inhibitors (**a**). These therapeutic ncRNAs can be delivered by some vectors, including PAE, PEG, lentivirus, adenovirus and plasmids, and can also be administered in the forms of ASO-lncRNAs, miR agomirs, and miR antagomirs to play a therapeutic role (**b**). ncRNA-based therapy has been shown to be effective in cell-derived xenograft (CDX) models, including subcutaneous, orthotopic and metastatic models (**c**). ncRNA-based therapy has been shown to be effective in patient-derived tumor xenograft (PDX) models (**d**). Lnc00958 sponges miR3619-5p in HCC, leading to the upregulation of hepatoma-derived growth factor and thereby promoting HCC adipogenesis and progression. In the PDX model, the weight and volume of the xenografts were significantly reduced after tail vein injection of PLGA-PEG (si-LINC00958) NPs (**e**).

Several ncRNA-based therapies, including siRNAs, shRNAs, sgRNAs, therapeutic lncRNAs, miRNA mimics, and miR inhibitors (Fig. 5a), have been shown to effectively suppress the malignant phenotype of cancer in mice. In recent years, new types of platforms, such as polymeric nanoparticle (NP) platforms and poly (β-amino esters) (PAEs), have been developed as carriers of cancer treatment that effectively deliver siRNA to target cells and tissues (Fig. 5b). The efficacy of targeting m6A effectors or their target ncRNAs for cancer treatment has been demonstrated in a variety of mouse models, including subcutaneous, orthotopic, metastatic and patient-derived tumor xenograft (PDX) models (Fig. 5c, d). For example, Lnc00958 sponges miR3619-5p in HCC, leading to the upregulation of hepatoma-derived growth factor and thereby promoting HCC adipogenesis and progression. In the PDX model, the weight and volume of the xenografts were significantly reduced after tail vein injection of PLGA-PEG (si-LINC00958) NPs (Page 20, Lines 565-569) (Fig. 5e)^40^. In a cell-derived xenograft (CDX) model, tail vein injection of local short hairpin RNA to stably knock down LCAT3 (shLACT3) was found to inhibit tumor growth in lung cancer^47^. In vivo delivery of circSORE RNAi via local short hairpin RNA lentiviral injection significantly enhanced the efficacy of sorafenib in animal models^61^. The growth of endometrioid endometrial carcinoma cells was inhibited in mice transplanted with pcDNA-FENDRR-transfected cells^155^. In a PDX model, tail vein injection of NEAT1–1 #4 m6A-mut had no positive effect on the distant metastasis of mouse prostate cancer^58^. Tumor growth was significantly suppressed by in vivo-optimized RNA interference (RNAi) inhibition of LINRIS^156^. PAEs to assist in the transport of circMDK siRNA (PAE-siRNA) were demonstrated to be effective in inhibiting tumor progression without causing significant adverse effects in four HCC models, including a subcutaneous model, orthotopic model, lung metastasis model and PDX model^134^. These studies suggest that targeting RNA effector molecules for clinical treatment is a promising research direction. However, in general, our understanding of ncRNAs and RNA modification is not thorough, and more studies are needed to help us explore more accurate and faster detection methods and to develop more effective targeted therapies.

## Other RNA chemical modifications

In addition to m6A modification, RNA can also undergo more than 170 chemical modifications, including m1A, m5C, m7G, ac4C, etc. m1A refers to the methylation of the first N atom of adenine in RNA molecules. Unlike m6A, m1A mainly dominates tRNA and rRNA, leading to changes in their secondary structure and playing a role in affecting translation^157,158^. m1A modifications can also occur in mRNAs and affect their stability and translation^159,160^. m5C is the methylation of the 5th carbon atom of cytosine. m5C is also involved in multiple RNA metabolisms, including mRNA export^161^, RNA stability^162^ and translation^163^. m7G refers to the methylation modification of the 7th N atom of guanine usually located at the 5’ cap of mRNA^164^, rRNA^165^, tRNA^166^ and miRNAs^167^. At present, a few studies have discussed m7G modification and revealed that m7G and other RNA epigenetic modifications have synergistic effects on different biological processes^168^. ac4C is the acetylation modification of the 4th N atom of cytosine, which was first discovered on tRNA and helps tRNA read codons correctly^169^. ac4C modification has also been demonstrated to exist on rRNA, which is related to maintaining the accuracy of translation^170^. ac4C also widely exists in mRNA and affects its stability^171^.

## Conclusion

In summary, the study of m6A effectors and their target ncRNAs provides new directions for the diagnosis and treatment of cancer. Although the mechanism underlying the effect of m6A modification on ncRNA metabolism has been well studied and progress has been made in research on the contribution of m6A effectors and related ncRNAs to tumor phenotypes, current treatments have only been validated in mouse models, and substantial work remains to render clinical translation feasible. This is the direction of future research on the m6A modification of ncRNAs.
